# Implementation, adherence, and results of systematic SARS-CoV-2 testing for asymptomatic patients treated at a tertiary care regional radiation oncology network

**DOI:** 10.1186/s13014-021-01760-2

**Published:** 2021-02-04

**Authors:** Roshan S. Prabhu, Reshika Dhakal, Amy S. Hicks, James McBride, Alicia L. Patrick, Christopher D. Corso, Tomain Murphy, Melissa Thonen, Edward H. Lipford, Derek Raghavan, Stuart H. Burri

**Affiliations:** 1 Levine Cancer Institute, Atrium Health, 1021 Morehead Medical Drive, Suite 1000, Charlotte, NC 28204 USA; 2 Southeast Radiation Oncology Group, Charlotte, NC USA

**Keywords:** Radiation oncology, Radiotherapy, COVID-19, SARS-cov-2

## Abstract

**Background:**

Coronavirus disease 2019 (COVID-19) caused by the SARS-CoV-2 virus is a current pandemic. We initiated a program of systematic SARS-CoV-2 polymerase chain reaction (PCR) testing in all asymptomatic patients receiving radiotherapy (RT) at a large radiation oncology network in the Charlotte, NC metropolitan region and report adherence and results of the testing program.

**Methods:**

Patients undergoing simulation for RT between May 18, 2020 and July 10, 2020 within the Levine Cancer Institute radiation oncology network who were asymptomatic for COVID-19 associated symptoms, without previous positive SARS-CoV-2 testing, and without recent high-risk contacts were included. PCR testing was performed on nasal cavity or nasopharyngeal swab samples. Testing was performed within 2 weeks of RT start (pre-RT) and at least every 4 weeks during RT for patients with prolonged RT courses (intra-RT). An automated task based process using the oncology electronic medical record (EMR) was developed specifically for this purpose.

**Results:**

A total of 604 unique patients were included in the cohort. Details on testing workflow and implementation are described herein. Pre-RT PCR testing was performed in 573 (94.9%) patients, of which 4 (0.7%) were positive. The adherence rate to intra-RT testing overall was 91.6%. Four additional patients (0.7%) tested positive during their RT course, of whom 3 were tested due to symptom development and 1 was asymptomatic and identified via systematic testing. A total of 8 (1.3%) patients tested positive overall. There were no known cases of SARS-CoV-2 transmission from infected patients to clinic staff and/or other patients.

**Conclusions:**

We detailed the workflows used to implement systematic SARS-CoV-2 for asymptomatic patients at a large radiation oncology network. Adherence rates for pre-RT and intra-RT testing were high using this process. This information allowed for appropriate delay in initiating RT, minimizing the occurrence of RT treatment interruptions, and no known cases of transmission from infected patients to clinic staff and/or other patients.

## Background

The severe acute respiratory syndrome coronavirus 2 (SARS-CoV-2) virus which causes the coronavirus disease 2019 (COVID-19) illness is a current pandemic both worldwide and in the United States [1]. Patients with cancer are considered at increased risk for severe COVID-19 illness [2] and have been shown to have elevated rates of COVID-19 mortality [3, 4]. The Levine Cancer Institute (LCI) of Atrium Health initiated a comprehensive program for care of patients with cancer during the COVID-19 pandemic, which included social distancing initiatives, telephone and in-person screening of patients for symptoms and temperature, visitor restrictions, expanded use of telemedicine care, systematic use of personal protection equipment (PPE) by both staff and patients, alternating staff schedules, and triaging care based on severity of the cancer condition [5]. As part of this approach, we initiated a program of systematic SARS-CoV-2 polymerase chain reaction (PCR) testing in all asymptomatic patients receiving radiotherapy (RT) at a large multicenter radiation oncology network in the Charlotte, NC metropolitan region. Implementation of this testing program required the establishment of novel processes utilizing automated task creation and workflows to identify patients, order PCR testing, follow-up on test results, and set reminders for ongoing PCR testing as set intervals.

Herein, we detail the processes and workflows used to implement systematic pre-radiotherapy (pre-RT) and intra-radiotherapy (intra-RT) SARS-CoV-2 PCR testing of asymptomatic patients receiving RT in a large regional radiation oncology network as well report the results and adherence to the testing program.

## Methods

All patients undergoing simulation for RT between May 18, 2020 and July 10, 2020 within the Levine Cancer Institute, Atrium Health radiation oncology network who were asymptomatic for COVID-19 associated symptoms [6], without previous positive SARS-CoV-2 testing, and without recent high risk contacts were eligible. The network consists of 9 radiation oncology centers, of which 2 were closed during the majority of the project time period as part of the pandemic response [5]. PCR testing was performed on nasal cavity or nasopharyngeal swab samples using the Roche (Basel, Switzerland) or Luminex (Austin, TX) platforms. Pre-RT testing was performed at the time of initial simulation and/or within 2 weeks prior to RT start. Patients who remained asymptomatic with prolonged RT courses were also retested systematically at least every 4 weeks during RT (intra-RT testing). Patients who developed COVID-19 related symptoms not explained by their treatment course were additionally tested. Project data were collected prospectively and were locked for analysis on September 15, 2020. This project was deemed to be IRB exempt as a quality improvement project.

**Fig. 1:**
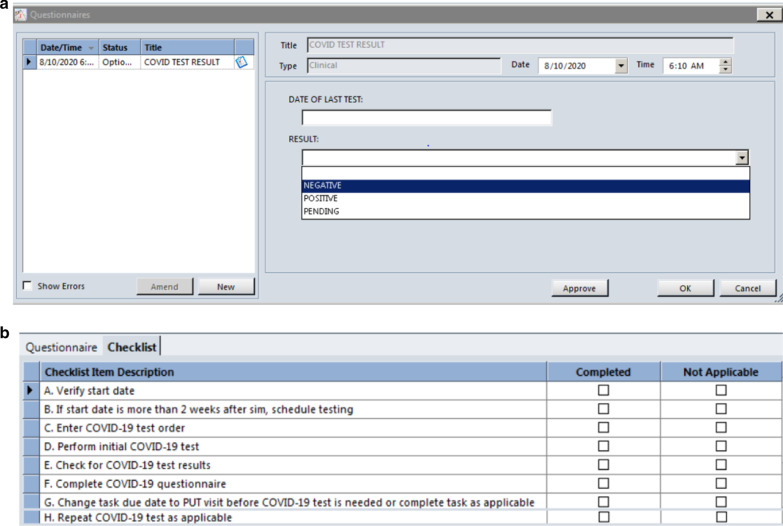
(**a**) SARS-CoV-2 results questionnaire example for a test patient in Aria. This questionnaire was used to record SARS-CoV-2 testing results in the Aria electronic medical record. Date of the last test was entered and the result was entered as a pulldown menu with options of negative, positive, or pending. (**b**) Nursing COVID-19 task checklist example for a test patient in Aria. The checklist was used to standardize the 8 steps (steps A–H) needed for both initial and ongoing SARS-CoV-2 test ordering and tracking

Eight of nine radiation oncology centers in the network use the Aria oncology information system (Varian medical systems, Palo Alto, CA) as the radiation oncology specific electronic medical record (EMR). A primary focus of this report will be the processes implemented utilizing the Aria EMR. The initial radiotherapy simulation (CT or clinical) was the encounter used as the starting point for systematic asymptomatic SARS-CoV-2 testing. Patients were contacted the day prior to their simulation to screen for COVID-19 related symptoms. When a CT or clinical simulation was scheduled in Aria, a COVID-19 nursing task was automatically created. The task consisted of 2 parts: a questionnaire and a checklist. The questionnaire was used to document PCR testing dates and results in the Aria system (Fig. 1a). The checklist was used to standardize the 8 steps needed for PCR ordering and tracking (Fig. 1b). The policy for pre-RT testing was to have a SARS-CoV-2 PCR test result documented within 2 weeks prior to RT start for asymptomatic patients.

After simulation, the patient would proceed to be seen by a nurse to determine if they had a recent prior negative PCR test that would satisfy the pre-RT testing policy. If a recent test had been performed, the nurse would enter the PCR testing date into the COVID-19 questionnaire and document the result in the pulldown menu (Fig. 1a). If no recent test had been performed within 2 weeks prior to RT start, the nurse would arrange for PCR testing. Prior to June 29, 2020, the nurse would enter orders for a test collection to be performed at a specialized testing site (i.e. drive up or tent site) and clerical staff would then schedule the test for the patient. The patient would leave the clinic with the appointment information for PCR testing in hand. Starting June 29, 2020, we began performing nasal cavity swab specimen collection in clinic and the nurse would perform the test for the patient immediately after the simulation if they were going to begin RT within 2 weeks. If the RT start date was more than 2 weeks away, the patient was scheduled for a future 15 min nursing appointment in the radiation oncology clinic that was labeled as “COVID-19 Test Collection” for a nasal cavity specimen collection approximately 1 week prior to RT start. The nurse would then enter the PCR testing date (either that day or future date) into the COVID-19 questionnaire and mark the result as pending from the dropdown menu (Fig. 1a). Additionally, the radiation therapist initial chart check encounter was modified to add a “COVID-19 test results” check as part of the initial chart check before RT start. This check was meant to confirm documentation of pre-RT SARS-CoV-2 status within the Aria questionnaire form.

The nursing COVID-19 task checklist (Fig. 1b) was used as an ongoing multi-part task within Aria to standardize the implementation and tracking of SARS-CoV-2 testing. The first 4 steps of the task (steps A–D) include activities described above, which would be checked off after completion. After performing or scheduling a test, the nurse would then change the due date of the task to 2 days after the date of the PCR test. The task would then come due to act as a reminder to check for the SARS-CoV-2 test results (step E) and to enter the results into the Aria COVID-19 questionnaire (step F). If the patient tested positive, the supervising radiation oncologist would be informed and the patient would be reflexively contacted by Atrium Health to assess symptoms, educate the patient about isolating and give other information, and determine if the patient needed to be admitted to the virtual hospital [5].

**Fig. 2:**
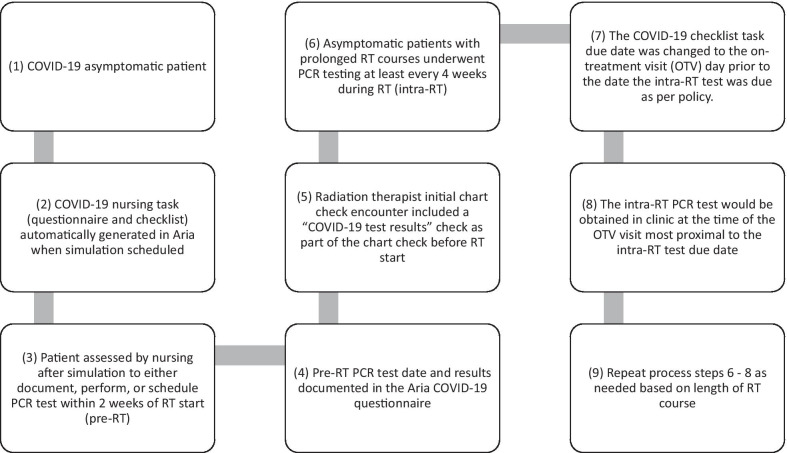
SARS-Cov-2 testing and tracking workflow using the Aria electronic medical record. PCR = polymerase chain reaction, RT = radiotherapy,

Intra-RT SARS-CoV-2 testing was performed at least every 4 weeks for patients who remained asymptomatic with prolonged RT courses (> 4 weeks). Intra-RT test 1 was defined as the test performed 4 weeks after the pre-RT test and intra-RT test 2 was defined as the test performed 4 weeks after intra-RT test 1. If the patient had a prolonged RT course, the nurse then would set the due date for the nursing COVID-19 task to the on treatment visit (OTV) day prior to the date the intra-RT test was due as per policy (4 weeks from the previous PCR test, step G). This task would serve as a reminder to obtain the intra-RT test on an at least every 4 week basis. The intra-RT PCR testing schedule was also reinforced by automatically importing and displaying the most recent PCR test date and result from the COVID-19 questionnaire into the weekly OTV note in Aria in order to have that information easily available on a weekly basis (Fig. 2).

Patients who developed COVID-19 related symptoms [6] not explained by their treatment course or who had high risk exposures were sent for nasopharyngeal SARS-CoV-2 PCR testing at designated testing sites. The general principles suggested by Filippi et al. (Priority 3) for patients with suspected or confirmed COVID-19 were employed, however the decision to delay RT start or interrupt ongoing treatment were made by consensus by a group of physicians based on multiple factors such as patient risk factors for severe COVID-19 outside of the cancer diagnosis, symptom status, specific cancer type and stage, and estimated consequence of a RT treatment break or delay [7]. If a patient had not yet started RT and was found to be asymptomatically positive (i.e. at the pre-RT test), it was policy to delay the start of RT if clinically appropriate due to uncertainty about whether the patient would remain asymptomatic or was actually pre-symptomatic. It was felt to likely be less detrimental to delay RT start in most cases rather than risk treatment interruptions if the patient became symptomatic. Patients who were deemed persons under investigation (PUI) or found to be SARS-CoV-2 positive who continued RT were treated using a standardized approach, including staff using full PPE including eye protection, N95 masks, and gowns; the patient being scheduled preferentially at the end of the day, not using common waiting rooms or dressing rooms, and using department side access if possible for entrance and exiting and proceeding directly to the treatment vault to minimize any unnecessary contact with staff or other patients.

## Results

North Carolina (NC) SARS-CoV-2 case rates were increasing over the project time period with 511 new cases reported on May 18, 2020 and 1982 new cases reported on July 10, 2020 [8]. The same increasing trend was also documented more locally within the region of analysis. A total of 604 unique patients were included in the cohort (Table 1). Most patients had breast (31%), prostate (18.2%), or lung cancer (17.7%). About 1/3 of patients had previous SARS-CoV-2 testing unrelated to RT, most of whom (79.4%) had a single negative previous test.

**Table 1:** Patient cohort characteristics (n = 604)

Variable	No. (%) or median (IQR)
*Gender*	
Male	274 (45.5%)
Female	330 (54.6%)
*Primary Cancer*	
Lung	107 (17.7%)
Breast	187 (31%)
Melanoma	8 (1.3%)
Renal cell carcinoma	7 (1.2%)
Gastrointestinal	42 (7%)
Gynecologic	17 (2.8%)
Prostate	110 (18.2%)
Lymphoma	6 (1%)
Myeloma	6 (1%)
Central nervous system	25 (4.1%)
Head & neck	42 (7%)
Sarcoma/connective tissue	14 (2.3%)
Non-melanoma skin cancer	20 (3.3%)
Leukemia	1 (0.2%)
Non-prostate Genitourinary	11 (1.8%)
Endocrine	1 (0.2%)
*Previous SARS-CoV-2 PCR test*	
Yes	204 (33.8%)
No	400 (66.2%)
*If previous SARS-CoV-2 test, number*	
1	162 (79.4%)
2	36 (17.6%)
3	6 (2.9%)
*If previous SARS-CoV-2 test, result*	
Negative	204 (100%)
*Systemic therapy within 3 months prior to RT*	
Yes	257 (42.5%)
No	347 (57.5%)
*Systemic therapy concurrent with RT*	
Yes	167 (27.6%)
No	437 (72.4%)
*Patient admission status*	
Inpatient	27 (4.5%)
Outpatient	577 (95.5%)
Age (years)	65 (56–73)
RT duration (weeks)	4 (1.6–6.1)
RT number of fractions	15 (5–25)

No. = number, IQR = interquartile range, PCR = polymerase chain reaction, RT = radiotherapy

Pre-RT PCR testing was performed in 573 patients (94.9%). Of the 31 patients (5.1%) who did not undergo pre-RT testing, 7 declined to be tested, 3 missed their off-site testing appointments, 1 had indeterminant test results, 1 did not have pre-RT testing ordered due to receiving a single fraction of RT, and 19 had no specific reason documented. Of the 573 patients who underwent pre-RT testing, 4 (0.7%) were positive (Table 2). One patient was an inpatient for non-COVID-19 related reasons and RT was initiated as planned due to urgent indications. The other 3 positive patients were outpatient and initiation of their planned RT courses was delayed between 19 and 38 days. Two of these 4 patients eventually developed COVID-19 related symptoms, 1 mild and 1 of moderate severity [9].

**Table 2:** SARS-CoV-2 positive patient characteristics (n = 8)

Case	Timing of positive test	Gender	Age (years)	Primary tumor type	RT intent	RT dose (Gy) / fractions	Symptom status at time of test	RT course
1	Pre-RT	Female	36	Melanoma	Palliative whole brain RT	30/10	A	Initiated RT as planned. Retested 14 and 16 days after initial positive test, both negative
2	Pre-RT	Female	79	Bladder	Palliative SBRT to lung oligometastasis	54/3	A	SBRT start held. Retested 19 days and 27 days later, both negative. SBRT start delayed 27 days
3	Pre-RT	Male	60	Prostate	Definitive RT + brachytherapy boost for unfavorable intermediate risk prostate cancer	46/23	A	RT start held. Retested 31 days and 33 days later, both negative. Patient resimulated due to delay. RT start delayed 38 days
4	Pre-RT	Female	52	Breast	Definitive breast + regional lymph node RT	50/25 + 10/5 boost	A	RT start held. Retested 14 and 16 days later, both negative. Patient resimulated due to delay. RT start delayed 19 days
5	Intra-RT	Female	51	Breast	Definitive breast RT	42.56/16 + 10/4 boost	S	Pre-RT test performed 4 days prior to intended RT start was negative. Patient’s husband tested positive and she became symptomatic 2 days prior to intended RT start. RT start held. Patient tested and was positive. Retested 15 days later and was positive again. Retested 15 and 18 days later, both negative. Patient started AI in the interim and RT start delayed 10 weeks
6	Intra-RT	Female	71	Breast	Definitive breast (DCIS) RT	42.56/16 + 10/4 boost	S	Pre-RT test negative. Patient initiated RT and received 6 of 20 planned fractions. She then became symptomatic and RT was held for 2 days for testing, which was positive. Repeat test 14 days later was also positive. 2^nd^ repeat test 2 week later was also positive. RT resumed after 5 week delay
7	Intra-RT	Male	62	Prostate	Definitive post-prostatectomy salvage RT for PSA failure	68.4/38	A	Pre-RT test negative. Intra-RT asymptomatic test positive after 25 fractions. RT held for 1 day and then resumed and completed. He was retested 28 and 30 days after initial positive test, both still positive
8	Intra-RT	Female	68	Breast	Definitive bilateral breast	50/25 + 10/5 boost	S	Pre-RT test negative. After 15 fractions, she became symptomatic and RT was held for 3 days for testing, which was positive. RT was resumed after the 3 day break and then completed as planned. Repeat test 8 days after initial positive test was also positive. 2^nd^ repeat test after an additional 10 days was negative. 3^rd^ repeat test after an additional 4 days was positive

RT = radiotherapy, SBRT = stereotactic body RT, PSA = prostate specific antigen, A = asymptomatic, S = symptomatic

The eligible population for intra-RT testing 1 consisted of 266 patients (44%), of which 247 (92.9%) underwent testing and 19 (7.1%) did not. No patients tested positive during asymptomatic systematic intra-RT testing 1. A total of 57 patients (9.4%) were eligible for intra-RT testing 2 due to prolonged RT courses, of which 49 (86%) underwent testing and 8 (14%) did not. One patient tested positive during intra-RT testing 2 (Case 7, Table 2), who completed RT as intended with a delay of 1 day. This patient did not develop symptoms and remained asymptomatic. Three additional patients were found to be positive during their RT course due to testing initiated by symptom development (Table 2). One patient had a RT break of 3 days, 1 patient had a RT break of 5 weeks, and 1 patient had her initial RT start delayed by 10 weeks. A total of 8 (1.3%) patients tested positive overall, of which 5 were asymptomatic and identified via systematic testing. Two of these 5 asymptomatic patients (40%) eventually developed symptoms, 1 of mild and 1 of moderate severity.

## Discussion

We have demonstrated the ability to perform systematic pre-RT and intra-RT SARS-CoV-2 testing in an asymptomatic population of patients undergoing RT at a large regional multi-center radiation oncology network. We developed a protocol and workflow using automated tasks, alerts, and checklists within the radiation oncology EMR that utilized pre-appointment patient screening for symptoms, was linked to RT simulation scheduling for initiation, and carried through the patient’s entire RT course. Though this protocol specifically used the Aria oncology information system, the principles and workflows laid out in this study can be applied to any particular radiation oncology EMR system. The process set forth here utilizes the entire radiation oncology team, including clerical staff, therapists, nurses, and physicians to ensure systematic ongoing PCR testing, follow-up, and action for positive test results.

Despite the de novo implementation of this system at the beginning of the study period (May 18, 2020), the adherence rates for pre-RT, intra-RT test 1, and intra-RT test 2 (if applicable) were high. Several patients did not undergo pre-RT testing due to missing their off site pre-RT PCR test appointments. This issue was addressed with the change to intra-department nasal cavity swab collection on the day of simulation starting June 29, 2020.

The overall rate of positivity in this patient population was low at 1.3% overall. The asymptomatic pre-RT testing positivity rate was 0.7% and asymptomatic intra-RT testing positivity rate was 0.2%. Three patients (0.5%) underwent additional testing due to symptom development and were found to be positive. This is despite significantly increasing community SARS-CoV-2 case incidence during the inclusion period both state-wide and locally [8]. The protocols instituted within the radiation oncology network were part of a larger approach to oncology care during the SARS-CoV-2 pandemic at the Levine Cancer Institute [5]. The low rates demonstrated here are likely the result of the combined effect of this overall approach, which included staff and patient masking/PPE requirements, visitor restrictions, patient and family education about safety during the pandemic, use of telemedicine appointments, screening for symptoms and temperature checks, and routine asymptomatic SARS-CoV-2 testing performed pre-procedure, for patients receiving immunosuppressive systemic therapy, and for patients undergoing RT amongst other efforts. Additionally, 43% of patients in this cohort received previous systemic therapy within 3 months of RT and approximately 1/3 had previous negative PCR testing, indicating interaction with the healthcare system, and the aforementioned protocols, upstream from their RT course, which could have led to reduced rates of SARS-CoV-2 positivity compared to newly diagnosed patients. The highest yield of asymptomatic testing occurred with pre-RT testing (0.7%), with only 1 additional patient testing positive via asymptomatic testing during intra-RT testing. Two of the 5 asymptomatic patients (40%) eventually developed symptoms, 1 mild and 1 moderate severity. These 2 patients were pre-symptomatic and their RT start was delayed, thereby likely avoiding a RT treatment interruption if their start had proceeded as initially planned. Most patients who tested positive during their RT course were due to symptom development.

Several of the SARS-CoV-2 positive patients in this cohort demonstrated persistently positive PCR tests despite asymptomatic status or resolution of previous symptoms. Persistent viral RNA positivity has been reported in the literature [10, 11]. Since the risk of viral replicative competency and viral transmission for these patient not well defined and patients with cancer represent a group with increased risk of COVID-19 morbidity and mortality [2–4], we adopted the approach of recommending quarantine and postponement of cancer treatment until the patient has demonstrated viral clearance using PCR testing, if possible. The results from this systemic SARS-CoV-2 testing program allowed for appropriate delay in initiating RT and minimizing the occurrence of RT treatment interruptions. Importantly, there were no known cases of viral transmission from infected patients to clinic staff and/or other patients under these protocols.

Limitations of this study include the potential for false negative test results [12], though nasopharyngeal/nasal cavity sample PCR testing represents the current standard of care for SARS-CoV-2 testing [13]. We also were not able to determine the specific reasons for non-adherence to testing for the majority of untested patients. Strengths of this study include the prospective study design, detailed description of process and workflow implementation and steps, large patient cohort from a multicenter regional radiation oncology network, and information on both pre-RT and intra-RT testing results as a measure of both initial and ongoing risk of viral infection during a patient’s RT course.

## Data Availability

The datasets generated and/or analysed during the current study are not publicly available due data use agreements and institutional policy.
